# Supplementary material for: Lateral Flow Immunoassay with Quantum-Dot-Embedded Silica Nanoparticles for Prostate-Specific Antigen Detection
Source: Nanomaterials (Basel). 2021 Dec 23;12(1):33. doi: 10.3390/nano12010033 (PMC8746978; doi:10.3390/nano12010033)
Supplement: Supplementary file 1 [file nanomaterials-12-00033-s001.zip › nanomaterials-1497369-supplementary.pdf]

nanomaterials

Article

Lateral Flow Immunoassay with Quantum-Dot-embedded Silica Nanoparticles for Prostate-Specific Antigen Detection

Sungje Bock[1], Hyung-Mo Kim[1,†], Jaehi Kim[1], Jaehyun An[1,2], Yun-Sik Choi[3], Xuan-Hung Pham[1], Ahla Jo[1], Kyeong-min Ham[1], Hobeom Song[2], Jung-Won Kim[2], Eunil Hahm[1], Won-Yeop Rho[4], Sang Hun Lee[5], Seung-min Park[6], Sangchul Lee[7], Dae Hong Jeong[3], Ho-Young Lee[8,*], and Bong-Hyun Jun[1,*]

1 Department of Bioscience and Biotechnology, Konkuk University, Seoul 05029, South Korea; bsj4126@konkuk.ac.kr (S.B.); hmkim0109@konkuk.ac.kr (H.-M.K.); susia45@gmail.com (J.K.); ghj4067@konkuk.ac.kr (J.A.); phamricky@gmail.com (X.-H.P.); iamara0421@konkuk.ac.kr (A.J.); hkm7321@konkuk.ac.kr (K.-m.H.); greenice@konkuk.ac.kr (E.H.)

2 BioSquare Inc, Hwaseong 18449, South Korea; hbsong@bio-square.com (H.S.); jwkim@bio-square.com (J.-W.K.)

3 Department of Chemistry Education, Seoul National University, Seoul 08826, South Korea; 71388c@naver.com (Y.-S.C.); jeongdh@snu.ac.kr (D.H.J.)

4 School of International Engineering and Science, Jeonbuk National University, Jeonju 54896, South Korea; rho7272@jbnu.ac.kr

5 Department of Chemical and Biological Engineering, Hanbat National University, Deajeon 34158, South Korea; sanghunlee@hanbat.ac.kr

6 Department of Urology, Stanford University School of Medicine, Palo Alto, CA 94305, United States; sp293@stanford.edu

7 Department of Urology, Seoul National University Bundang Hospital, Seongnam 13620, South Korea; slee@snubh.org

8 Department of Nuclear Medicine, Seoul National University Bundang Hospital, Seongnam 13620, South Korea

* Correspondence: debobkr@gmail.com (H.-Y.L.); bjun@konkuk.ac.kr (B.-H.J.); Tel.: +82-31-787-2938 (H.-Y.L.); +82-2-450-0521 (B.-H.J.)

† Current address: KIURI Research Center, Ajou University, Suwon 16499, Korea.

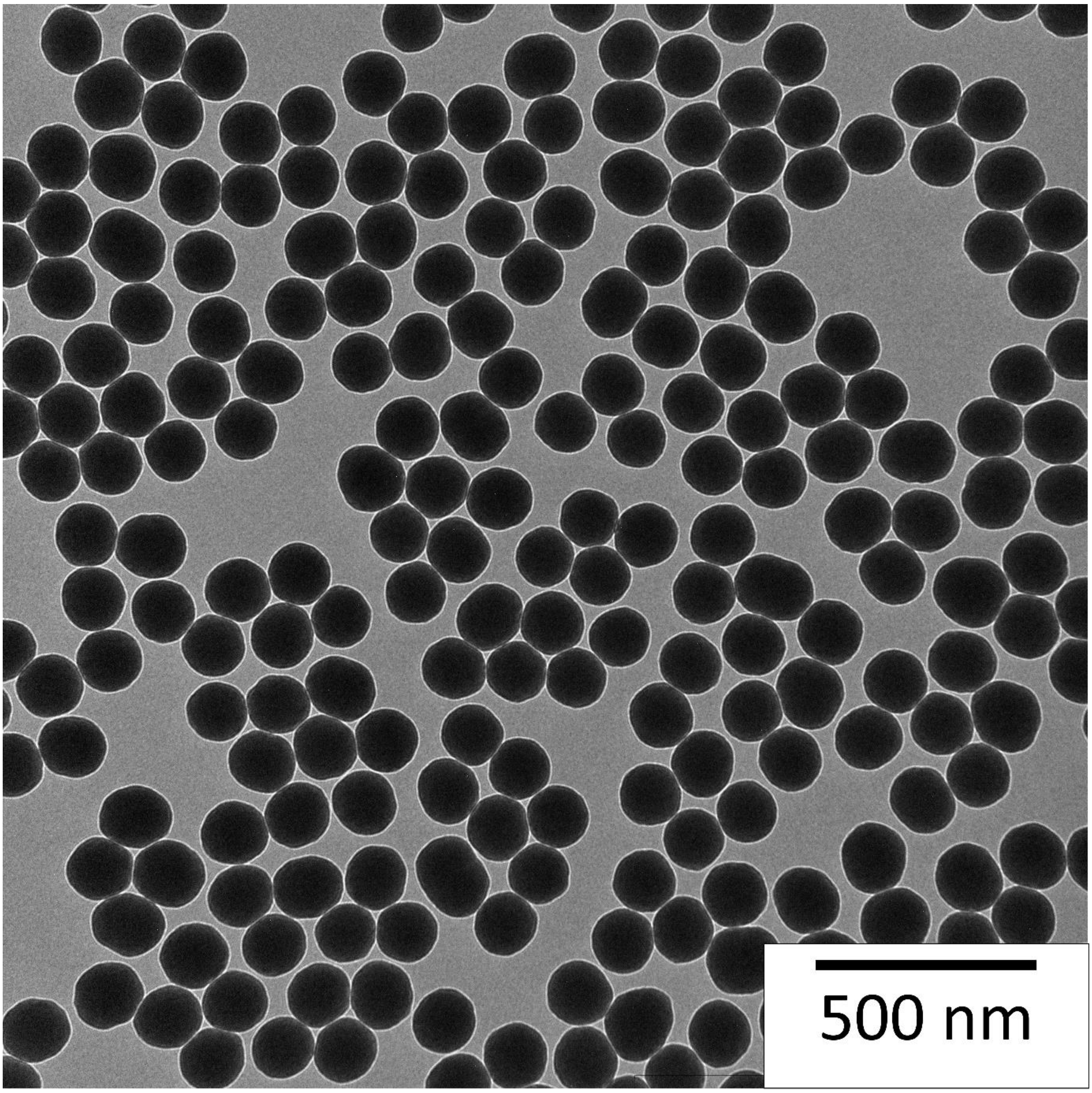

Figure S1. TEM image of SiO_2 NPs.

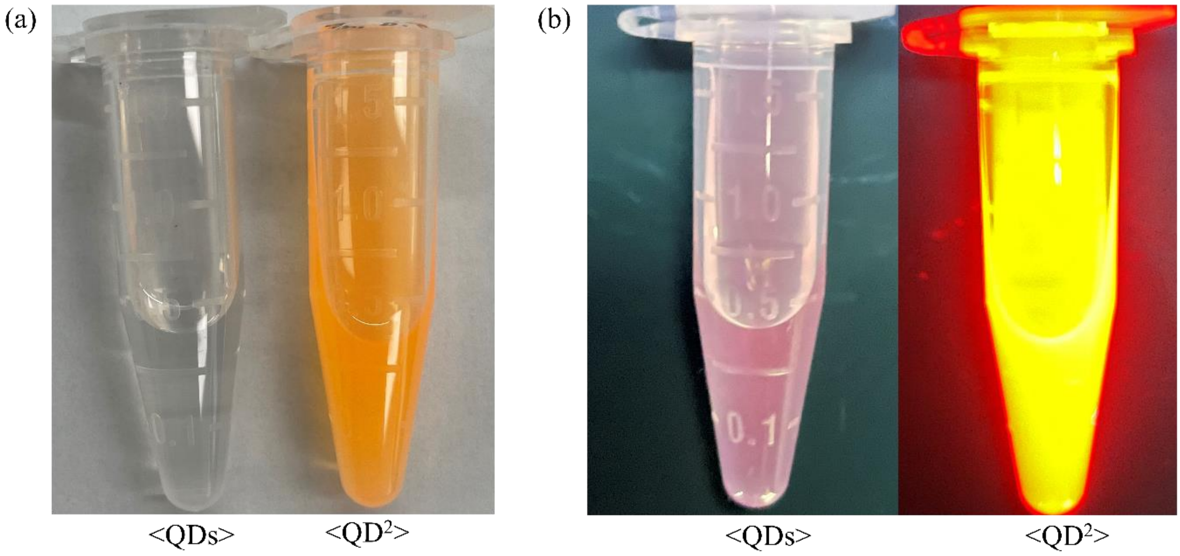

Figure S2. Comparison of fluorescence intensity between QDs and QD². Images of QDs and QD² (**a**) under visible light and (**b**) UV (365 nm) light.

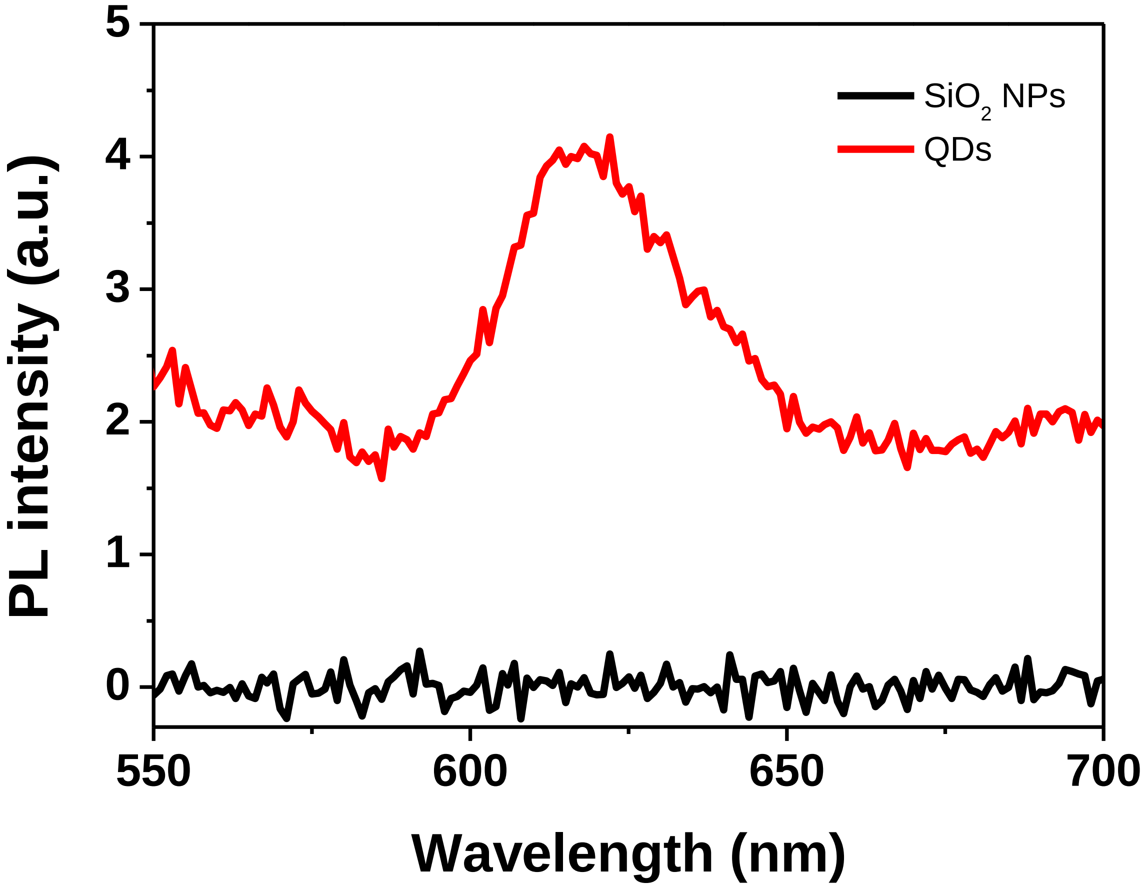

Figure S3. PL intensity of QDs.

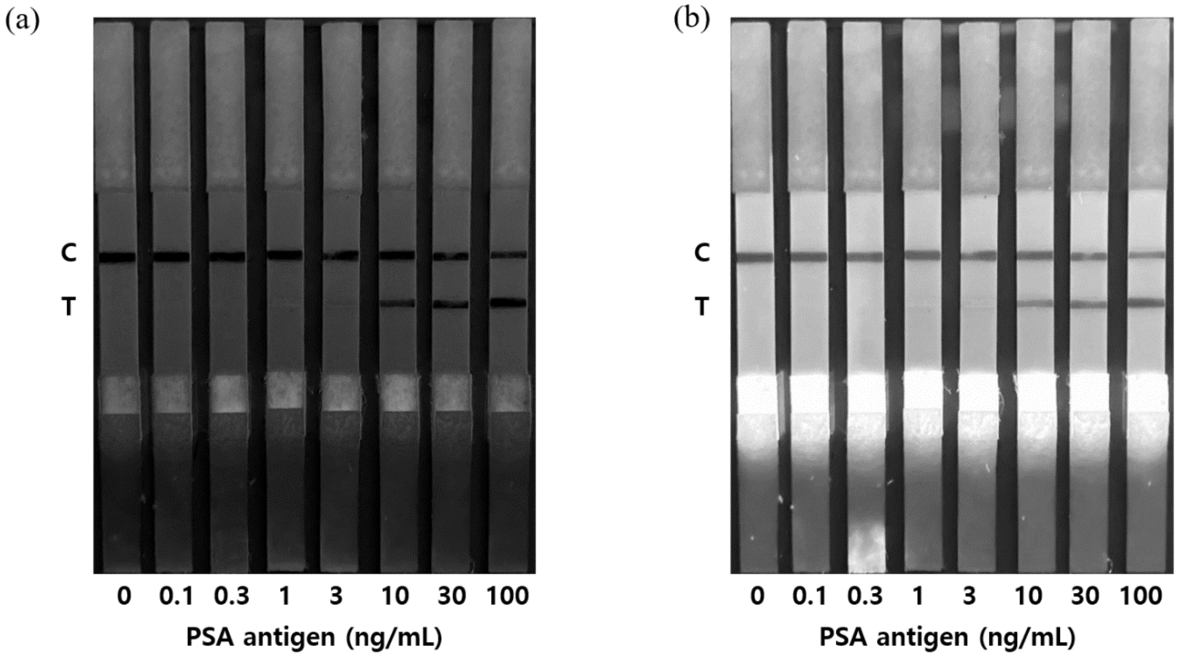

Figure S4. Fluorescence image of the test strip with the (**a**) green and (**b**) blue channels separated with PSA developed under a 365-nm UV lamp.

$$y = 1.03993 - 1.01744 \times 0.86081^{x}$$

Figure S5. Equation of PSA detection fitting curve shown in Fig 2c.
